# Supplementary material for: The footprint of column collapse regimes on pyroclastic flow temperatures and plume heights
Source: Nat Commun. 2019 Jun 6;10:2476. doi: 10.1038/s41467-019-10337-3 (PMC6554404; doi:10.1038/s41467-019-10337-3)
Supplement: Supplementary file 1 — Supplementary Information [file 41467_2019_10337_MOESM1_ESM.pdf]

Supplementary Information

The footprint of column collapse regimes on pyroclastic flow temperatures and plume heights

Trolese et al.

Supplementary Figures

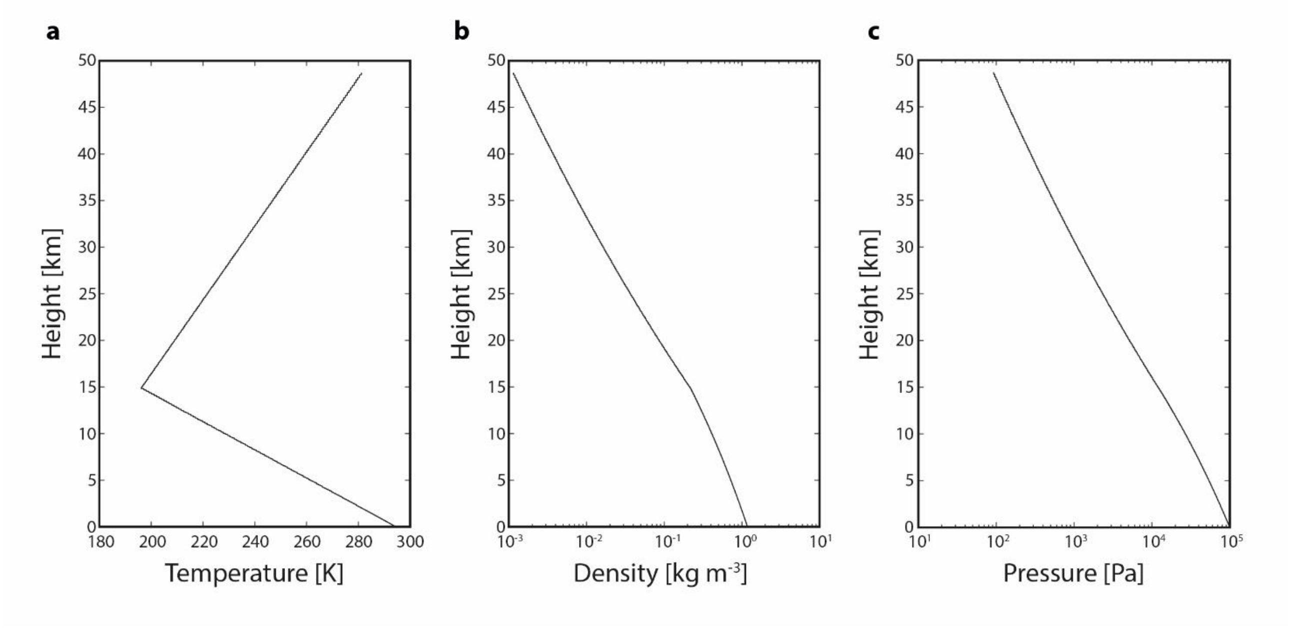

Supplementary Figure 1 | Atmospheric profiles used for the simulations. a Temperature, **b** density, and **c** pressure as functions of the height above sea level.

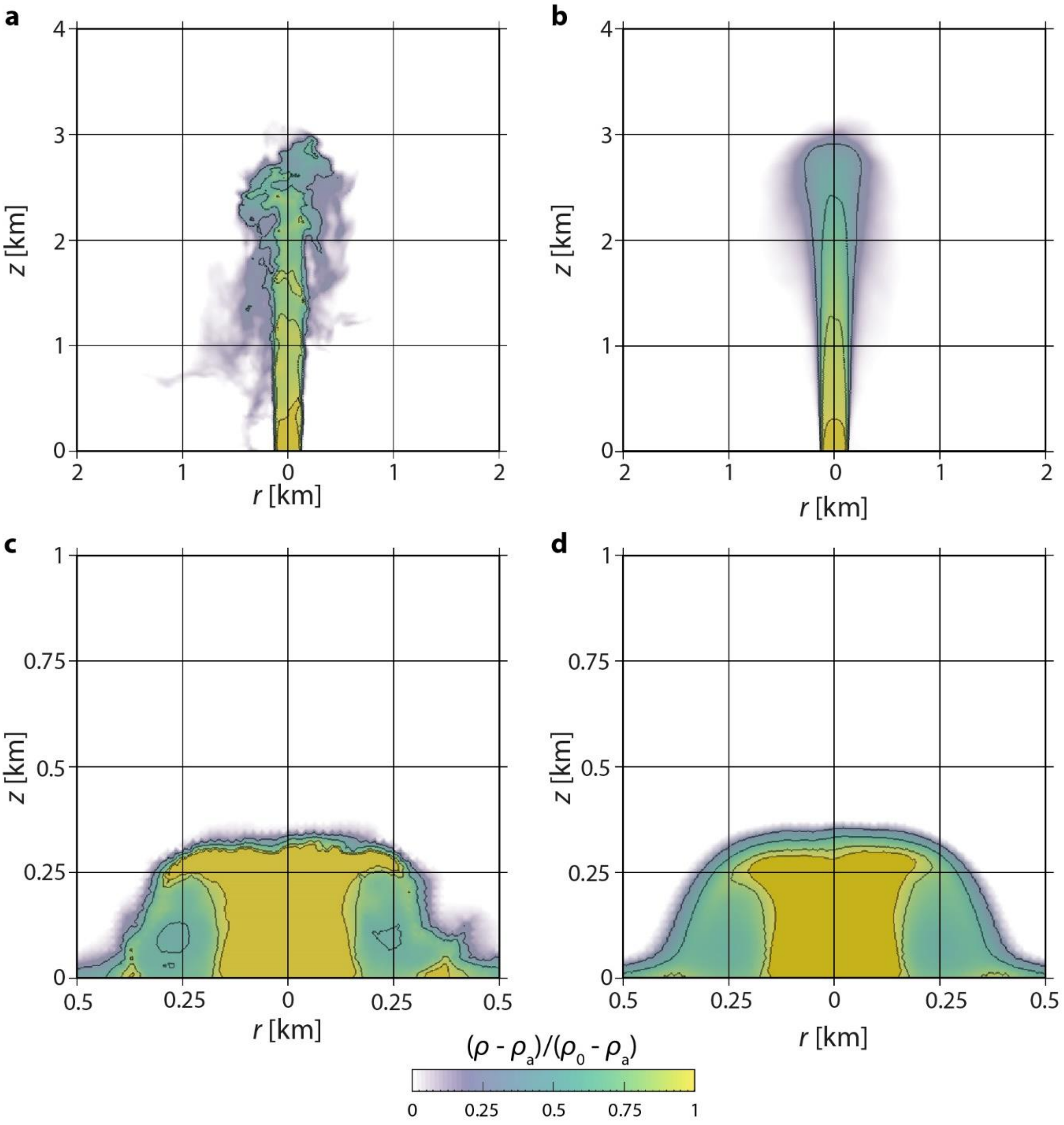

Supplementary Figure 2 | Comparison between the internal structures of the two column collapse end-members. a, c, Cross-sectional images showing the instantaneous distribution of the density difference relative to the stratified atmospheric density at the same vertical position normalized to the initial density, at 100 s and 315 s respectively, for the partial (**a**) and total (**c**) collapse. Isolines correspond to $\rho - \rho_a / \rho_0 - \rho_a = 0.25, 0.50, 0.75, 0.99$. **b, d**, Same as **a, c**, but in a time-averaged domain. The averaging window is from 500 to 1000 s.

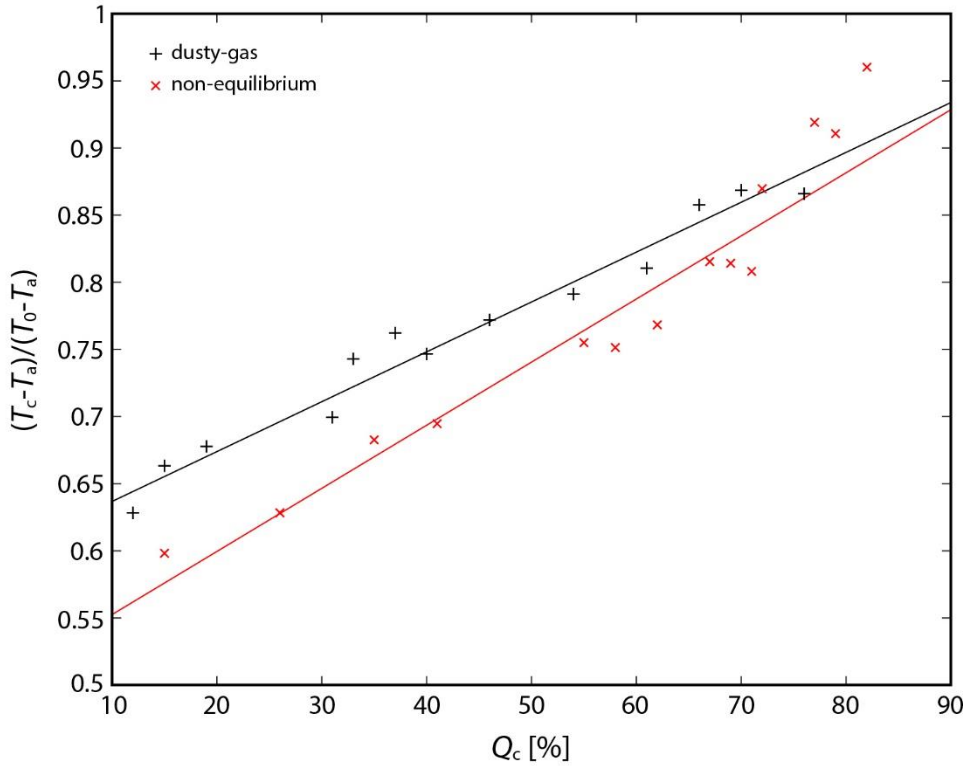

Supplementary Figure 3 | Comparison between dusty-gas and kinematic decoupling simulations. Relationship between the percentage of collapsing mass Q_c and its corresponding average temperature value T_c normalized to the initial magmatic temperature T_0 with respect to the atmospheric air temperature T_a, for dusty-gas (black symbols) and kinematic decoupling (red symbols) simulations with the same initial eruptive conditions (mass eruption rate Q = 10^8 kg s^{-1}; temperature T = 1123 K; water content $0.5<y_w<2.5$ wt%; pressure $0.1<P<10$ MPa). The black and red solid lines show the linear least square regression for dusty-gas and kinematic decoupling simulations, respectively. The red regression line is drawn using all data from kinematic decoupling simulations (it is the same shown in Figure 3c).

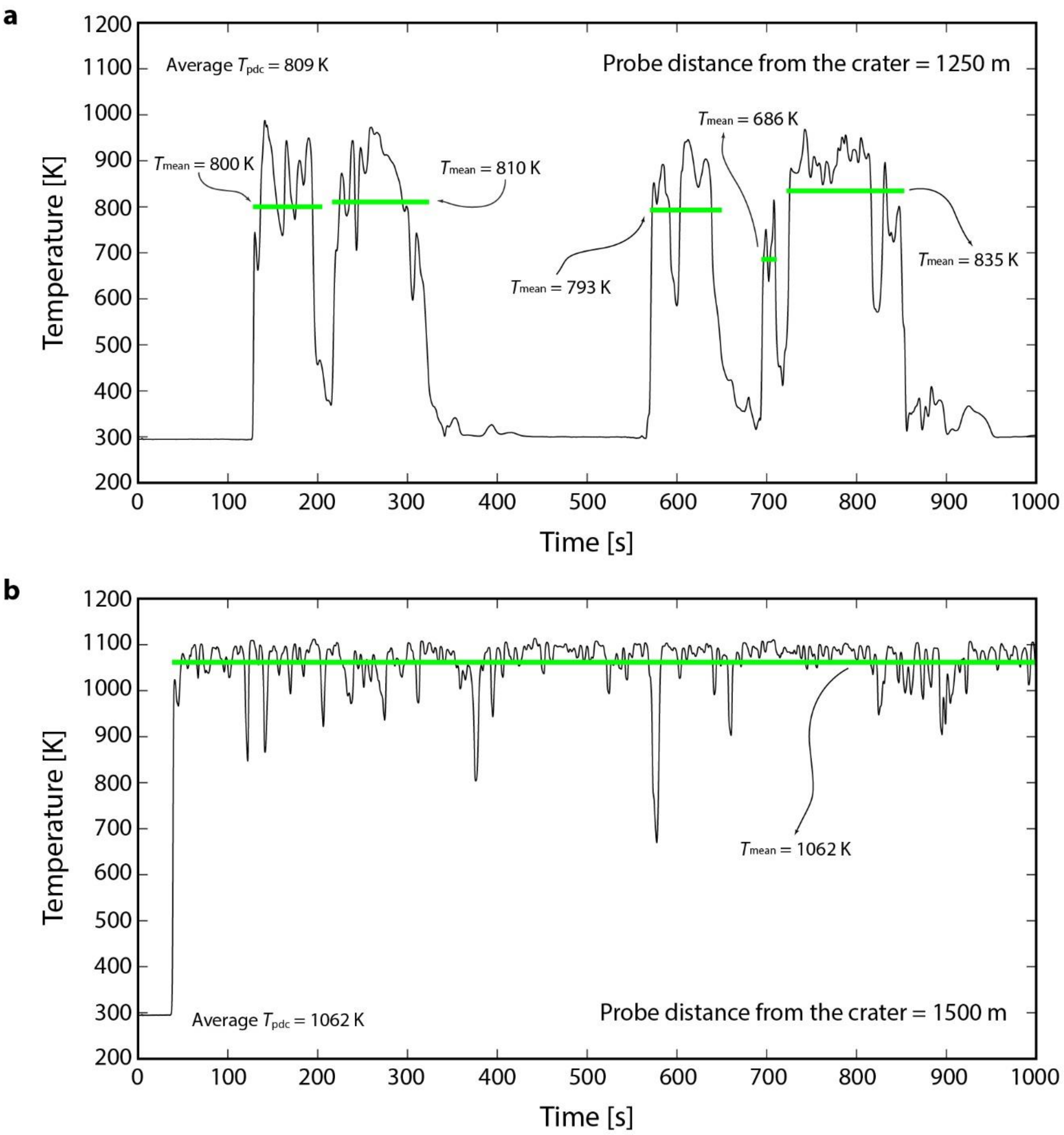

Supplementary Figure 4 | Time series of PDC temperature data for the reference models. Temporal evolution of the mixture temperature (black solid line) in the bottom layer of PDC generated by the partial (**a**) and total (**b**) column collapse end-members. Temperature is measured at a distance from the injection point of 10 inlet radii ($r = 10r_0$). Green solid lines indicate the time-averaged temperature of each pulse (T_{mean}). The PDC temperatures (T_{pdc}) averaged over the reported time-window are 809 K and 1062 K for the partial and total collapse end-members, respectively. Such temperatures are very similar (within less than 5% in this case) to the mean temperatures at collapse.

Supplementary Tables

Supplementary Table 1 | Input parameters used for the 3D simulations and collapse properties. All notation is given in the Methods section. Bold characters represent the end-members described in the main text. The independent variables are given at the conduit exit (vent level). The remaining dependent variables at the vent have the subscript $_v$, while those at the inlet level – i.e., after the decompression – have the subscript $_0$. A choked condition is imposed at the conduit exit, i.e., the exit velocity of the mixture is the speed of sound.

Independent variables				Dependent variables						Q_c	T_c
Q (kg s^{-1})	T (K)	y_w (wt.%)	P (MPa)	ρ_v (kg m^{-3})	U_v (m s^{-1})	r_v (m)	ρ_0 (kg m^{-3})	U_0 (m s^{-1})	r_0 (m)	(%)	(K)
10^7	923	2.0	0.1	11.72	92.3	54.2	11.7	92.3	54.2	65	767
10^7	1123	1.0	0.1	19.27	72.0	47.9	19.3	72.0	47.9	70	945
10^7	1123	1.5	0.1	12.84	88.2	53.0	12.8	88.2	53.0	59	900
10^7	1123	2.0	0.1	9.647	102	56.9	9.65	102	56.9	41	857
10^7	1123	2.5	0.1	7.709	113	60.2	7.71	113	60.2	24	804
10^7	1123	1.0	0.2	38.54	72.0	33.8	19.3	108	39.1	53	889
10^7	1123	1.5	0.2	25.69	88.2	37.5	12.8	132	43.3	18	756
10^7	1123	2.0	0.2	19.27	102	40.3	9.64	153	46.5	-	-
10^7	1123	2.5	0.2	15.41	114	42.6	7.71	171	49.2	-	-
10^7	1123	1.5	10	1284	88.2	5.29	12.8	175	37.6	-	-
10^7	1123	2.5	10	770.9	114	6.02	7.71	226	42.7	-	-
10^7	1273	1.0	0.1	17.00	76.7	49.4	17.0	76.7	49.4	64	1056
10^7	1273	2.0	0.1	8.501	108	58.7	8.50	108	58.7	24	933
10^7	1273	2.5	0.1	6.801	121	62.1	6.80	121	62.1	-	-
10^8	1123	0.5	0.1	38.54	50.9	127	38.5	50.9	127	82	1090
**10^8**	**1123**	**1.0**	**0.1**	**19.27**	**72.0**	**151**	**19.3**	**72.0**	**151**	**79**	**1049**
10^8	1123	1.5	0.1	12.84	88.2	167	12.8	88.2	167	72	1015
10^8	1123	2.0	0.1	9.637	102	180	9.64	102	180	67	970
10^8	1123	2.5	0.1	7.709	114	190	7.71	114	190	62	931
10^8	1123	0.5	0.2	77.09	50.9	90.0	38.5	76.4	104	77	1056
10^8	1123	1.0	0.2	38.54	72.0	107	19.3	108	123	71	964
10^8	1123	1.5	0.2	25.69	88.2	118	12.8	132	137	55	920
10^8	1123	2.0	0.2	19.27	102	127	9.64	153	147	41	870
10^8	1123	2.5	0.2	15.42	114	134	7.71	171	155	26	815
10^8	1123	0.5	10	3854	50.9	12.7	38.5	101	90.3	69	969
10^8	1123	1.0	10	1927	72.0	15.1	19.3	143	107	58	917
10^8	1123	1.5	10	1284	88.2	16.7	12.8	175	118	35	860
**10^8**	**1123**	**2.0**	**10**	**964**	**102**	**18.0**	**9.64**	**203**	**127**	**15**	**790**
10^8	1123	2.5	10	771	114	19.0	7.71	226	135	-	-
10^8	1273	1.0	0.1	17.00	76.7	156	17.0	76.7	156	74	1158
10^8	1273	1.0	0.2	34.00	76.7	110	17.0	115	127	62	1059
10^8	1273	2.0	0.2	17.00	108	131	8.50	163	152	24	951
10^8	1273	1.5	10	1133	93.9	17.3	11.3	187	123	10	836
10^9	1123	3.0	0.1	6.425	124	630	6.42	124	630	74	1009
10^9	1123	4.0	0.1	4.818	144	677	4.82	144	677	57	939
10^9	1123	5.0	0.1	3.855	161	716	3.85	161	716	36	855
10^9	1123	6.0	0.1	3.212	176	749	3.21	176	749	18	785
10^9	1123	3.0	0.2	12.85	124	445	6.42	187	514	42	885
10^9	1123	4.0	0.2	9.637	144	478	4.82	216	552	16	773

Q_c: percentage of collapse, T_c: average temperature of the collapsing mass

Supplementary Table 2 | Thermodynamic constants used for the simulations

Gas constant of water vapor (R_{w}; J kg^{-1}K^{-1})	462
Gas constant of air (R_{air}; J kg^{-1}K^{-1})	287
Specific heat at constant pressure of air ($C_{P\mathrm{air}}$; J kg^{-1} K^{-1})	1004
Specific heat at constant pressure of water ($C_{P\mathrm{w}}$; J kg^{-1}K^{-1})	1810
Specific heat solid particles (C_{s}; J kg^{-1}K^{-1})	1100
Density of coarse ash particles (kg m^{-3})	2200
Density of fine ash particles (kg m^{-3})	2700

Supplementary Table 3 | Collection of emplacement temperature data. Emplacement temperatures of some column collapse pyroclastic flow deposits, their bulk volumes and their related eruption temperatures are taken from the literature. These data are visualized in Fig. 4. Pyroclastic flow deposits that are associated with phreatomagmatic eruptions and dome collapses were not included in the table. For those pyroclastic flow deposits where a bulk volume is not reported in the literature, we assigned a deposit volume that is lower than the total volume of the erupted material. Values denoted by * are given by using the typical eruption temperatures based on melt composition. We provide only a qualitative description of welded pyroclastic flow deposits, and hence we do not assign them a specific eruption temperature (we denoted these cases with #).

Location – Eruption	V (km^3)	Deposit temperature	Eruption temperature	Authors
Athesian Volcanic Group – 274 Ma Ora	~ 1290	Welded	#	ref. [1]
Cerro Galán – 2.08 Ma Cerro Galán	~ 630	≥620°C/welded	~800°C	refs. [2–4]
Cerro Galán – 4.2 Ma Real Grande	510	≥600°C	~800°C	refs. [2–4]
Colima – 1913 AD F5	<0.26	280 – 330°C	~860°C	refs. [5–7]
Colima – 1913 AD F4	<0.26	250 – 300°C	~860°C	refs. [5–7]
Colli Albani – 0.35 Ma Villa Senni	30	≥630°C	~1100°C	refs. [8,9]
Colli Albani – 0.40 Ma Pozzolane Nere	15	≥600°C	~1100°C	refs. [8,9]
Colli Albani – 0.45 Ma Pozzolane Rosse	60	≥630°C	~1100°C	refs. [8,9]
El Chicón – 1982 AD	<1.1	360 – 420°C	~800°C	refs. [6,10,11]
Fogo – 4.6 ka Fogo A (Pink intra-plinian ignimbrite)	<1.2	350 – 400°C	~900°C	ref. [12]
Fogo – 4.6 ka Fogo A (Black intra-plinian ignimbrite)	<1.2	580 – 620°C	~900°C	ref. [12]
Fogo – 4.6 ka Fogo A (Dark-brown ignimbrite)	<1.2	250 – 370°C	~900°C	ref. [12]
Great Basin ignimbrite province – 22.93 Ma Pahranagat Formation	2100	Welded	#	refs. [13,14]
Great Basin ignimbrite province – 24.95 Ma Clipper Gap Tuff	180	Densely welded	#	ref. [14]
Great Basin ignimbrite province – 26.82 Ma Hancock Tuff	1100	Densely welded	#	ref. [14]
Great Basin ignimbrite province – 26.98 Ma Coyote Summit Tuff	1400	Welded	#	ref. [14]
Great Basin ignimbrite province – 27.57 Ma Monotony Tuff	4500	Welded	#	ref. [14]
Great Basin ignimbrite province – 29 Ma Lund Tuff	~ 3000	Welded	#	ref. [15]
Great Basin ignimbrite province – 29.97 Ma Palisade Mesa Tuff	200	Welded	#	ref. [14]
Great Basin ignimbrite province – 31.69 Ma Windous Butte ignimbrite	4800	Densely welded	#	ref. [14]
Great Basin ignimbrite province – 35.3 Ma Pancake Summit Tuff	700	Densely welded	#	ref. [14]
Krakatau – 1883 AD	26.7	475 – 550°C	~900°C*	refs. [16,17]
Lascar – 1993 AD	0.4	≥397°C	~900°C*	refs. [18,19]
Long Valley caldera – 0.76 Ma Bishop Tuff	500	Welded	#	refs. [20,21]
Mt. St. Helens – 22 July 1980	0.006	≥580°C	~920°C	refs. [19,22]
Mt. St. Helens – 12 June 1980	0.02	361 – 602°C	~920°C	refs. [22,23]
Mt. St. Helens – 12 June 1980	0.02	510 – 570°C	~920°C	refs. [19,22]
Mt. St. Helens – 18 May 1980	0.12	300 – 420°C	~920°C	refs. [22,23]
Montserrat – 26 December 1997	~ 0.09	48 – 293°C	~1000°C	ref. [24]
Montserrat – 21 October 1997	~ 0.019	400 – 560°C	~1000°C	refs. [24–26]
Montserrat – 17 October 1997	~ 0.019	180	~1000°C	refs. [24,25]
Montserrat – 17 September 1997	0.123	326	~1000°C	refs. [24,25]
Montserrat – 31 May 1997	0.003	99 – 149°C	~1000°C	refs. [24,25]
San Juan Volcanic field – 27.8 Ma Fish Canyon Tuff	5000	Welded	#	ref. [27]
Santorini – 18.5 ka Cape Riva breccias	>20	310 – 360°C	~880°C	refs. [28,29]
Santorini – 79 ka Middle Pumice	<14	250 – 350°C	~880°C	refs. [28,30]
Taupo – 1.8 ka	31.5	400 – 500°C	~800°C*	refs. [31,32]
Toba – 0.84 Ma Older Toba Tuff	~ 2500	Welded	#	refs. [33,34]
Toba – 75 ka Younger Toba Tuff	2800	Unwelded to welded	#	refs. [33,34]
Vesuvius – 1631 AD	0.19	380 – 400°C	~950°C	refs. [35–37]
Vesuvius – 472 AD Pollena	<0.01	260 – 340°C	~950°C	refs. [37,38]
Vesuvius – 79 AD (EU2/3pf; EU3pfi)	<0.75	220 – 280°C	~950°C	refs. [37,39,40]
Vesuvius – 79 AD (EU3pf)	<0.75	240 – 360°C	~950°C	refs. [37,39,40]
Vesuvius – 79 AD (EU3pfl)	<0.75	250 – 310°C	~950°C	refs. [37,39,40]
Vesuvius – 79 AD (EU2/3pf)	<0.75	258 – 371°C	~950°C	refs. [37,39,41]
Vesuvius – 79 AD (EU3pf)	<0.75	315 – 377°C	~950°C	refs. [37,39,41]
Vesuvius – 79 AD (EU3pf)	<0.75	140 – 300°C	~950°C	refs. [37,39,42]
Vesuvius – 9.5 ka Mercato	0.25	360 – 420°C	~950°C	refs. [35–37]
Vesuvius – 22 ka Pomici di Base	~ 0.18	340 – 380°C	~950°C	refs. [35–37]
Yellowstone – 0.6 Ma Lava Creek Tuff	1000	Densely welded	#	refs. [43,44]
Yellowstone – 2 Ma Huckleberry Ridge Tuff	2500	Densely welded	#	refs. [43,44]

References

1. Willcock, M. A. W. & Cas, R. A. F. Primary welding and crystallisation textures preserved in the intra-caldera ignimbrites of the Permian Ora Formation, northern Italy: implications for deposit thermal state and cooling history. *Bull. Volcanol.* **76,** 819 (2014).
2. Folkes, C. B. *et al.* A re-appraisal of the stratigraphy and volcanology of the Cerro Galán volcanic system, NW Argentina. *Bull. Volcanol.* **73,** 1427–1454 (2011).
3. Lesti, C. *et al.* High-temperature emplacement of the Cerro Galán and Toconquis Group ignimbrites (Puna plateau, NW Argentina) determined by TRM analyses. *Bull. Volcanol.* **73,** 1535–1565 (2011).
4. Folkes, C. B., Shanaka, de S. L., Wright, H. M. & Cas, R. A. F. Geochemical homogeneity of a long-lived, large silicic system; evidence from the Cerro Galán caldera, NW Argentina. *Bull. Volcanol.* **73,** 1455–1486 (2011).
5. Saucedo, R. *et al.* Eyewitness, stratigraphy, chemistry, and eruptive dynamics of the 1913 Plinian eruption of Volcán de Colima, México. *J. Volcanol. Geotherm. Res.* **191,** 149–166 (2010).
6. Sulpizio, R., Zanella, E., Macias, J. L. & Saucedo, R. Deposit temperature of pyroclastic density currents emplaced during the El Chichon 1982 and Colima 1913 eruptions. *Geol. Soc. London, Spec. Publ.* **396,** 35–49 (2014).
7. Macias, J., Arce, J., Sosa, G., Gardner, J. E. & Saucedo, R. Magma storage conditions of historic Plinian eruptions of Volcán de Colima, México. in *AGU Fall Meeting Abstracts* (2013).
8. Giordano, G. *et al.* The Colli Albani mafic caldera (Roma, Italy): Stratigraphy, structure and petrology. *J. Volcanol. Geotherm. Res.* **155,** 49–80 (2006).
9. Trolese, M., Giordano, G., Cifelli, F., Winkler, A. & Mattei, M. Forced transport of thermal energy in magmatic and phreatomagmatic large volume ignimbrites: Paleomagnetic evidence from the Colli Albani volcano, Italy. *Earth Planet. Sci. Lett.* **478,** 179–191 (2017).
10. Carey, S. & Sigurdsson, H. The 1982 eruptions of El Chichón volcano, Mexico (2): Observations and numerical modelling of tephra-fall distribution. *Bull. Volcanol.* **48,** 127–141 (1986).
11. Luhr, J. F. Experimental Phase Relations of Water- and Sulfur- Saturated Arc Magmas and the 1982 Eruptions of El Chichon Volcano. *J. Petrol.* **31,** 1071–1114 (1990).
12. Pensa, A., Giordano, G., Cas, R. A. F. & Porreca, M. Thermal state and implications for eruptive styles of the intra-Plinian and climactic ignimbrites of the 4.6 ka Fogo A eruption sequence, São Miguel, Azores. *Bull. Volcanol.* **77,** 99 (2015).
13. Best, M. G., Christiansen, E. H., Deino, A. L., Grommé, C. S. & Tingey, D. G. Correlation and emplacement of a large, zoned, discontinuously exposed ash flow sheet: The 40Ar/39Ar chronology, paleomagnetism, and petrology of the Pahranagat Formation, Nevada. *J. Geophys. Res. Solid Earth* **100,** 24593–24609 (1995).
14. Best, M. G. *et al.* The 36–18 Ma Central Nevada ignimbrite field and calderas, Great Basin, USA: Multicyclic super-eruptions. *Geosphere* **9,** 1562–1636 (2013).
15. Maughan, L. L. *et al.* The Oligocene Lund Tuff, Great Basin, USA: a very large volume monotonous intermediate. *J. Volcanol. Geotherm. Res.* **113,** 129–157 (2002).

16. Mandeville, C. W., Carey, S., Sigurdsson, H. & King, J. Paleomagnetic evidence for high-temperature emplacement of the 1883 subaqueous pyroclastic flows from Krakatau volcano, Indonesia. *J. Geophys. Res.* **99,** 9487–9504 (1994).

17. Crosweller, H. S. *et al.* Global database on large magnitute explosive volcanic eruptions (LaMEVE). *J. Appl. Volcanol.* **1,** 1–13 (2012).

18. Bernard, D., Oscar Figueroa, A., Eduardo Medina, T., Jose Viramonte, G. & Mario Maragaño, C. Petrology of pumices of April 1993 eruption of Lascar (Atacama, Chile). *Terra Nov.* **8,** 191–199 (1996).

19. Paterson, G. A. *et al.* Paleomagnetic determination of emplacement temperatures of pyroclastic deposits: an under-utilized tool. *Bull. Volcanol.* **72,** 309–330 (2010).

20. Sheridan, M. F. Particle-Size Characteristics of Pyroclastic Tuffs. *J. Geophys. Res.* **76,** 5627–5634 (1971).

21. Wilson, C. J. N. & Hildreth, W. Assembling an Ignimbrite: Mechanical and Thermal Building Blocks in the Bishop Tuff, California. *J. Geol.* **111,** 653–670 (2003).

22. Rutherford, J., Sigurdsson, H., Carey, S. & Davis, A. The May 18, 1980, Eruption of Mount St . Helens. 1. Melt composition and Experimental Phase Equilibria. *J. Geophys. Res.* **90,** 2929–2947 (1985).

23. Banks, N. G. & Hoblitt, R. P. *Direct Temperature Measurements of Deposits, Mount St. Helens, Washington, 1980-1981. U.S. Geol. Survey Prof. Paper* (1996).

24. Sparks, R. S. J. *et al.* Generation of a debris avalanche and violent pyroclastic density current on 26 December (Boxing Day) 1997 at Soufrière Hills Volcano, Montserrat. *Geol. Soc. London, Mem.* **21,** 409–434 (2002).

25. Cole, P. D. *et al.* Deposits from dome-collapse and fountain-collapse pyroclastic flows at Soufrière Hills Volcano, Montserrat. *Geol. Soc. London, Mem.* **21,** 231–262 (2002).

26. Scott, A. C. & Glasspool, I. J. Charcoal reflectance as a proxy for the emplacement temperature of pyroclastic flow deposits. *Geology* **33,** 589–592 (2005).

27. Steven, T. A. & Lipman, P. W. *Calderas of the San Juan Volcanic Field, Southwestern Colorado*. (USGS Prof. Pap. 958, 1975).

28. McClelland, E. A. & Druitt, T. H. Palaeomagnetic estimates of emplacement temperatures of pyroclastic deposits on Santorini, Greece. *Bull. Volcanol.* **51,** 16–27 (1989).

29. Fabbro, G. N., Druitt, T. H. & Scaillet, S. Evolution of the crustal magma plumbing system during the build-up to the 22-ka caldera-forming eruption of Santorini (Greece). *Bull. Volcanol.* **75,** 767 (2013).

30. Druitt, T. H. *et al.* Santorini Volcano. *Geol. Soc. Mem.* **19,** (1999).

31. Wilson, C. J. N. Stratigraphy, chronology, styles and dynamics of late Quaternary eruptions from Taupo volcano, New Zealand. *Phil. Trans. R. Soc. Lond. A* **343,** 205–306 (1993).

32. McClelland, E., Wilson, C. J. N. & Bardot, L. Palaeotemperature determinations for the 1.8-ka Taupo ignimbrite, New Zealand, and implications for the emplacement history of a high-velocity pyroclastic flow. *Bull. Volcanol.* **66,** 492–513 (2004).

33. Diehl, J. F., Onstott, T. C., Chesner, C. A. & Knight, M. D. No short reversals of Brunhes Age recorded in the Toba tuffs, north Sumatra, Indonesia. *Geophys. Res. Lett.* **14,** 753–756

(1987).

34. Rose, W. I. & Chesner, C. A. Dispersal of ash in the great Toba eruption, 75 ka. *Geology* **15,** 913–917 (1987).

35. Gurioli, L. *et al.* Pyroclastic flow hazard assessment at Somma–Vesuvius based on the geological record. *Bull. Volcanol.* **72,** 1021–1038 (2010).

36. Zanella, E., Sulpizio, R., Gurioli, L. & Lanza, R. Temperatures of the pyroclastic density currents deposits emplaced in the last 22 kyr at Somma-Vesuvius (Italy). *Geol. Soc. London, Spec. Publ.* **396,** 13–33 (2014).

37. Cioni, R., Marianelli, P. & Santacroce, R. Temperature of Vesuvius magmas. *Geology* **27,** 443–446 (1999).

38. Zanella, E., Gurioli, L., Lanza, R., Sulpizio, R. & Bontempi, M. Deposition temperature of the AD 472 Pollena pyroclastic density current deposits, Somma-Vesuvius, Italy. *Bull. Volcanol.* **70,** 1237–1248 (2008).

39. Cioni, R., Santacroce, R. & Sbrana, A. Pyroclastic deposits as a guide for reconstructing the multi-stage evolution of the Somma-Vesuvius Caldera. *Bull. Volcanol.* **61,** 207–222 (1999).

40. Cioni, R., Gurioli, L., Lanza, R. & Zanella, E. Temperatures of the A.D. 79 pyroclastic density current deposits (Vesuvius, Italy). *J. Geophys. Res. Solid Earth* **109,** B02207 (2004).

41. Caricchi, C., Vona, A., Corrado, S., Giordano, G. & Romano, C. 79 AD Vesuvius PDC deposits' temperatures inferred from optical analysis on woods charred in-situ in the Villa dei Papiri at Herculaneum (Italy). *J. Volcanol. Geotherm. Res.* **289,** 14–25 (2014).

42. Zanella, E., Gurioli, L., Pareschi, M. T. & Lanza, R. Influences of urban fabric on pyroclastic density currents at Pompeii (Italy): 2. Temperature of the deposits and hazard implications. *J. Geophys. Res. Solid Earth* **112,** B05214 (2007).

43. Christiansen, R. L. Yellowstone magmatic evolution: Its bearing on understanding large-volume explosive volcanism. *Explos. Volcanism Inception, Evol. Hazards* 84–95 (1984).

44. Christiansen, R. L. *The Quaternary and Pliocene Yellowstone Plateau volcanic field of Wyoming, Idaho, and Montana*. (2001).
